# Comparison of Treatment Outcome between Repetitive Transcranial Magnetic Stimulation (rTMS) and Transcutaneous Direct Current Stimulation (tDCS) in Intractable Tinnitus

**DOI:** 10.3390/jcm10040635

**Published:** 2021-02-07

**Authors:** Seong Hoon Bae, Seo Jin Moon, Jeong Gum Lee, Yun Kyung Yim, Hee So Oh, Dong Hee Han, In Seok Moon

**Affiliations:** 1 Department of Otorhinolaryngology, Yonsei University College of Medicine, Seoul 03722, Korea; bshsap@naver.com (S.H.B.); JINMOON31@yuhs.ac (S.J.M.); EDULJKC@yuhs.ac (J.G.L.); DBS1225@yuhs.ac (Y.K.Y.); happynews79@yuhs.ac (H.S.O.); 2 Yonsei Charm Otorhinolaryngology Clinic, Seoul 02490, Korea; superbrand@naver.com

**Keywords:** tinnitus, transcranial magnetic stimulation, transcranial direct current stimulation

## Abstract

Repetitive transcranial magnetic stimulation (rTMS) and transcutaneous direct current stimulation (tDCS) are non-invasive treatments for chronic tinnitus based on neuromodulation of cortical activity. Both are considered effective, but with heterogeneous results due to lack of established protocols. Because the target groups for both modalities overlap, it is difficult to recommend one of them. We tried to unify the inclusion criteria and treatment schedules to compare the two modalities. The medical charts of 36 patients who underwent rTMS as part of clinical routine were reviewed and data for 34 patients who underwent tDCS about 7 years later were collected prospectively. Both groups had chronic unilateral tinnitus refractory to medication. Patients were treated for 5 consecutive days, and tinnitus symptoms were evaluated by survey both at the end of the treatment schedule and 1 month after the treatment. The ratio of responders who showed >20% reduction in tinnitus handicap inventory scores were compared. At the end of the treatment, the rTMS group showed a rapid response compared to the tDCS group (rTMS, 30.6%; tDCS, 12.1%; *p* = 0.054). However, both groups showed a significant and similar reduction in tinnitus symptoms 1 month after the treatment (rTMS, 47.2%; tDCS, 36.4%; *p* = 0.618). As both groups showed comparable results for tinnitus reduction, tDCS may be superior in terms of cost-effectiveness.

## 1. Introduction

Tinnitus is a common acoustic problem which occurs in up to 21% of adults in their lifetime [1]. It is represented by phantom hearing without any external stimulation. In some patients, this can be explained by a maladaptive plastic brain that results from hearing impairment [2]. However, tinnitus still has many factors to be elucidated, including treatment. Although tinnitus does not seem to deteriorate hearing itself, tinnitus has significantly jeopardized psychological health in previous reports [3]. Nevertheless, evidence of a generally accepted treatment modality for tinnitus is still lacking.

Several non-invasive and neuromodulative treatments for reducing symptoms of tinnitus have been suggested and reported. For this, repetitive transcranial magnetic stimulation (rTMS) and transcranial direct current stimulation (tDCS) are used, which are based on neuro-modulation of brain cortical activity by electromagnetic field and direct current stimulation, respectively [4,5,6]. It is generally accepted that tinnitus patients exhibit neuronal hyperactivity in the central auditory pathways and co-activation of other structures, including the dorsolateral prefrontal cortex (DLPFC) [7,8]. rTMS and tDCS can change spontaneous neuronal firing and result in reduced tinnitus symptoms [9,10]. Previously, we reported long-term benefits of rTMS without major complications in patients with unilateral tinnitus [11,12]. Evidence of the effectiveness of tDCS for reducing tinnitus symptoms has also been established in several studies and systematic reviews [1,13,14,15]. In addition, tDCS is less expensive, easier to perform, and safer than rTMS [13].

Many studies have reported the effectiveness of tDCS and rTMS in controlling tinnitus severity. However, studies comparing the results of the two treatments are lacking. Given that neuromodulation therapy has shown variable effectiveness, comparing the results of both treatments in a single study with the same sampling criteria and unified treatment schedule can shed light on their different characteristics. Hence, the present study reports short-term and 1-month follow-up results of rTMS and tDCS at a single tertiary hospital.

## 2. Materials and Methods

Patients: A total of 70 patients were enrolled in this study: 36 underwent rTMS as part of our institution clinical routine for treatment of refractory chronic tinnitus from August 2012 to January 2013 and their medical charts were retrospectively reviewed for the purpose of the present study. Thirty-four patients prospectively underwent tDCS in the present study from February 2019 to October 2020. Moreover, 16 of the patients included in the rTMS protocol were included in our previous studies [11,12]. The same inclusion criteria were applied to both treatment groups: (1) unilateral chronic subjective tinnitus (tinnitus handicap inventory (THI) > 38, >6 months), and (2) tinnitus symptoms refractory to more than 2 months of medication (Gingko biloba and/or selective serotonin reuptake inhibitor). The exclusion criteria were as follows: (1) acute onset (<6 months), (2) pulsatile or clicking tinnitus, and (3) disease in the central nervous system. One patient withdrew from treatment because of a severe headache after the third tDCS session and was excluded from the study. Consequently, 33 patients were finally enrolled in the tDCS group. Informed consent was obtained from all prospectively enrolled patients. This study was approved by the institutional review board of Severance Hospital (1-2019-0040).

rTMS protocol: rTMS of the temporoparietal junction was performed for 5 days. Stimulation was applied to the left hemisphere based on previous reports that stimulation laterality does not impact the result and that left temporoparietal cortex stimulation is effective irrespective of tinnitus laterality [11,16,17]. A MagPro TMS unit connected to a Medtronic C-B65 figure-of-eight coil was used to deliver the stimulation (Magstim, Whitland, UK). The resting motor threshold (RMT) of the motor cortex was determined for the opposite-side abductor digiti minimi. The RMT was defined as the lowest intensity that evoked motor-evoked potentials of 50 mV in at least five of 10 stimuli. All patients underwent sham stimulation once before the first rTMS treatment. The patients were asked about the difference in tinnitus symptoms right after sham stimulation. For sham stimulation, the coil was placed in the same location at an angle of 90° to the scalp for 5 min. In this way, it generated a clicking sound of stimulation without actually stimulating the auditory cortex. None of the 36 patients enrolled in this study reported marked differences immediately after the sham stimulation. rTMS intensity of 90% of the RMT, with a rate of 1 Hz for 600 pulses (10 min; 8 s on, 2 s off) per session was applied once a day for 5 consecutive days.

tDCS protocol: Saline-soaked sponge electrodes were placed over the skin of the bilateral DLPFC area. The negative electrode (cathode) was placed on the left DLPFC area, and the positive electrode (anode) was placed on the right DLPFC area. Direct current was generated using the DC-STIMULATOR PLUS (neuroConn GmbH, Ilmenau, Germany). The patients underwent 10 min of tDCS per day for 5 consecutive days. Stimulation was continuous during the session with 1 mA intensity direct current. Patients were instructed to report any pain in the skin under the electrodes. No patients complained about skin pain, but one patient developed a severe headache after the third treatment and was excluded from the study.

Hearing and tinnitus evaluation: In both the rTMS and tDCS groups, patients were evaluated using pure tone audiogram, visual analog scale (VAS) loudness, VAS awareness, VAS annoyance, and tinnitus handicap inventory surveys before treatment. PTA was defined as the average pure tone threshold of 500 Hz, 1000 Hz, 2000 Hz, and 4000 Hz frequencies in the pure tone audiogram. Surveys including VAS loudness, VAS awareness, VAS annoyance, and THI were repeated immediately after the last session of treatment and 1 month after treatment. Improvement was defined as a >20% reduction in the THI score from the initial THI score.

Statistical analysis: Categorical variables were evaluated using a two-tailed Fisher’s exact test. Because some variables did not show normality, we adopted non-parametric statistical methods. Continuous variables were evaluated using Mann–Whitney’s U test if the compared groups were independent. For comparing serial changes in continuous variables for the same patients, a Friedman test and post-hoc Dunn’s test were used. The values in all figures and tables are presented as the mean ± SD. All statistical analyses were performed using IBM SPSS Statistics for Macintosh (version 24.0; IBM SPSS Statistics; IBM, Armonk, NY, USA), and a *p*-value less than 0.05 was considered statistically significant.

## 3. Results

### 3.1. Demographics of Enrolled Patients

Sixty-nine of the 70 patients were analyzed and divided into two groups according to treatment modality. Thirty-six patients underwent rTMS (21 men, 15 women) and 34 patients underwent tDCS and one was excluded because of an adverse event (headaches) and therefore 33 were analyzed (17 men, 16 women). The mean ages of the rTMS and tDCS groups were 56.1 ± 12.3 and 59.3 ± 11.2 years, respectively. The patients’ sex, age, and tinnitus side were not significantly different between the two groups. There were also no significant differences between the two groups in hearing function in the better and worse ear. Pre-treatment THI score and VAS score subcategories (loudness, awareness, and annoyance) were not significantly different between the two groups (Table 1).

### 3.2. Result of rTMS Immediately after Treatment and 1 Month after Treatment

**Figure 1:**
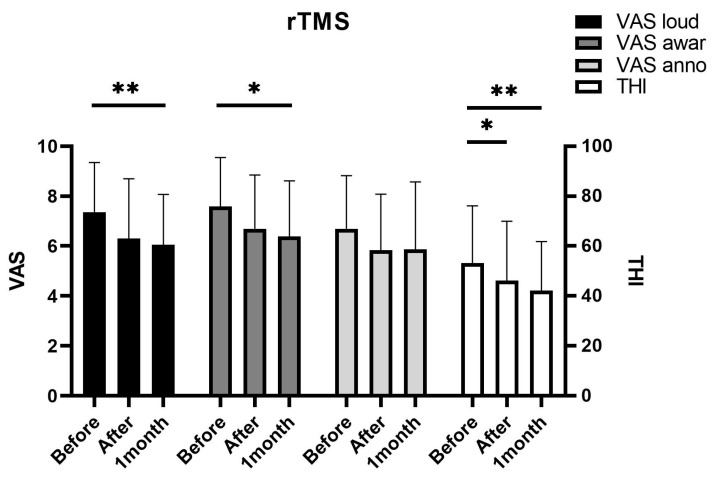
Repetitive transcranial magnetic stimulation (rTMS) rapidly reduces tinnitus symptoms. The rTMS group showed a rapid reduction of tinnitus symptoms evaluated by the visual analog scale and tinnitus handicap inventory survey immediately after treatment and sustained until 1 month after treatment. (* *p* < 0.05; ** *p* < 0.01; analyzed using repeated measure analysis of variance). VAS: visual analog scale; THI: tinnitus handicap inventory.

The THI and VAS scores tended to decrease after rTMS treatment (Figure 1). The Friedman test revealed that changes in all scores except VAS annoyance (*p* = 0.068) were statistically significant. After 1 month, VAS loudness (*p* = 0.005), VAS awareness (*p* = 0.012), and THI score (*p* = 0.001) showed significant improvements. However, VAS annoyance (*p* = 0.178) showed no significant improvement at 1 month after treatment. THI scores significantly reduced right after treatment (*p* = 0.040), and other scores tended to decrease right after treatment without statistical significance. Notably, none of the survey scores showed a significant change from immediately after to 1 month after treatment. All patients completed the rTMS sessions without any complications, including pain, headache, or hearing loss. Taken together, the effect of rTMS seemed to appear immediately after treatment and was sustained for at least 1 month.

### 3.3. Result of tDCS Immediately after Treatment and 1 Month after Treatment

**Figure 2:**
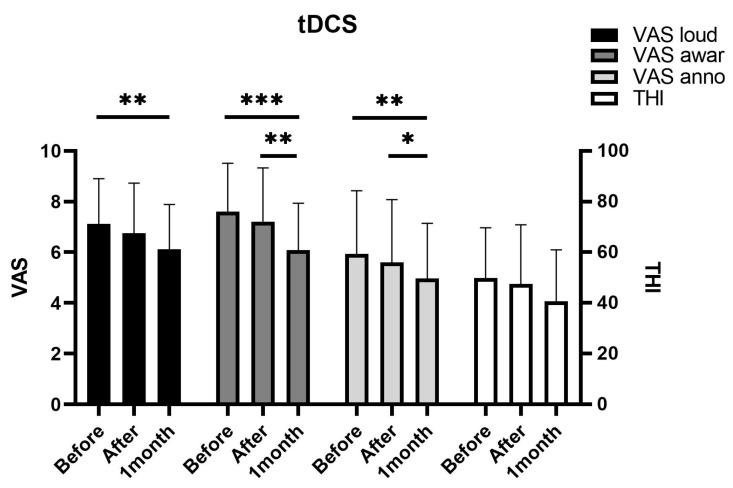
Transcranial direct current stimulation (tDCS) reduces tinnitus symptoms. The tDCS group showed reduction of tinnitus symptoms evaluated by the visual analog scale and tinnitus handicap inventory survey at 1 month after treatment. A significant reduction was noted between immediately after the treatment and 1 month after the treatment. (* *p* < 0.05; ** *p* < 0.01; *** *p* < 0.001; analyzed using repeated measure analysis of variance).

The Friedman test showed that all VAS subcategories were significantly different between time points. Post-hoc analysis showed all of the VAS subcategories (loudness, *p* = 0.003; awareness, *p* < 0.001; annoyance, *p* = 0.006) significantly improved at 1 month after tDCS (Figure 2). THI score (*p* = 0.058) also showed some improvement at 1 month after tDCS, although the difference lacked statistical significance. The Friedman test, however, revealed a significant difference (*p* = 0.031) therein between time points. Interestingly, none of the survey scores showed a significant improvement immediately after treatment. VAS scores were significantly improved at 1 month after treatment compared to those obtained immediately after treatment. Taken together, the effects of tDCS seemed to appear gradually after treatment. One patient withdrew from treatment because of a severe headache after the treatment and was excluded from the study. None of the remaining enrolled patients who completed tDCS sessions reported any complications, including skin injuries or headaches.

### 3.4. Comparison of Treatment Results between rTMS and tDCS

After statistical analysis, the results of the two modalities did not show any differences in VAS subcategories or THI scores (Table 2). Nevertheless, there seemed to be a tendency for rTMS to show rapid symptom improvement compared to tDCS as shown in paired-analysis. rTMS and tDCS showed improvement rates (more than 20% reduction in THI score) of 47.2% and 36.4%, respectively, which were not significantly different. Two patients after rTMS and three patients after tDCS showed worsening (more than 20% increase in THI score) of tinnitus symptoms 1 month after treatment.

## 4. Discussion

The rTMS and tDCS treatment protocols have not been standardized and the results of these modalities are very heterogeneous between studies. A systematic review and meta-analysis of seven randomized controlled trials for rTMS reported a THI reduction of 4–18 points and a response rate of 46.2% at 1 month after treatment [18]. Vanneste et al. reported that 29.9% to 46.67% of tinnitus patients responded to DLPFC area tDCS treatment (right anode, left cathode) decrease VAS score [19,20]. Frank et al. reported that DLPFC area tDCS treatment (right anode, left cathode) reduced VAS but not THI [21]. The result of tDCS stimulation on the left DLPFC (left anode, right cathode) were reportedly not significantly different from that on the right DLPFC (right anode, left cathode) in a randomized double-blinded control study by Faber et al. [14]. On the contrary, Vanneste et al. reported no tinnitus suppression effect for tDCS on the left DLPFC (left anode, right cathode) in 448 tinnitus patients [19]. Accordingly, placing an anode on the right DLPFC appears to be a more effective tDCS method, and the present study adopted this protocol.

The results of the present study showed that both rTMS on the left temporoparietal junction and tDCS of the right DLPFC were effective in treating tinnitus, which is refractory to medication, and resulted in improvement in both VAS and THI. We also compared the two treatment modalities at both scales and obtained more comprehensive results compared to previous studies. In our study, rTMS tended to elicit a more rapid response than tDCS. This implies that rTMS may be more effective in transient tinnitus reduction. Because both groups of patients were treated with the same schedule for five consecutive days, the temporal difference in treatment response seems more evident. However, the rapid tinnitus suppression effect of tDCS has been previously reported. Vanneste et al. reported that the tDCS effect remains for a mean duration of 1 d from immediately after 20 min of tDCS stimulation [6]. Although the difference was not statistically significant, the tDCS results of the present study also showed reduced mean scores in both VAS and THI immediately after treatment. Thus, the rapid response of tDCS should not be underestimated, and the exact mechanism of this difference should be elucidated in future studies by quantifiable cortical activity data.

Sustained effects of tinnitus suppression of more than 1 month have been reported for both rTMS and tDCS [22,23]. Our results also showed sustained effects of rTMS and tDCS until 1 month after treatment. In addition, the two treatment groups showed no significant differences at the 1-month time point, which implies that both treatments have comparable sustained tinnitus suppression effects. Regarding complications, rTMS has been found to provoke occasional seizures and, more commonly, headaches in up to 30% of subjects [24,25]. Patients also can experience discomfort during RMT measuring. On the other hand, there are no reports that tDCS is related to seizures, and the major reported adverse effects in other studies were pain and skin burns underneath the electrodes [13,26]. Headaches also can develop during and after tDCS treatment, although the incidence thereof is about 15%, according to the study by Kessler et al. [27]. The tDCS device application was easier because of the need to measure RMT immediately before every rTMS session started. The cost of the treatment device was lower for tDCS than for rTMS. The tDCS device used in the present study costs approximately $14,000, and the rTMS decide costs $80,000. In the present study, tDCS showed a comparable sustained effect on tinnitus suppression compared to rTMS. When considering the cost and reported risk of rTMS, tDCS clearly showed an advantage in terms of cost-effectiveness and safety.

The main limitation of the present study was that the treatment protocol may not be the most effective. Although we tried to unify the treatment schedule and inclusion criteria for subjects, the treatment effectiveness may differ according to the treatment protocols, as many previous studies have reported. Nevertheless, the present study showed similar results between the two treatment modalities, and the clinical application advantage of tDCS, which may inform clinicians when selecting a neuromodulation therapy for tinnitus. Second, a placebo control group was not included. In particular, the tDCS group did not conduct a sham protocol, unlike the rTMS group. Although the tinnitus suppression effect of sham and real tDCS seems to be distinctive based on previous studies, the absence of sham tDCS could significantly bias results [13,14,15,28]. Thus, the significance of the present study has to be limited to the approachability of clinical use, not to the superiority of the treatment efficacy. A well-designed randomized control trial should be performed to definitively compare the treatment efficacy of rTMS and tDCS in the future.

## 5. Conclusions

In the present study, rTMS was likely more effective than tDCS for transient tinnitus reduction. One month after treatment, both groups showed comparable results for tinnitus reduction. Thus, for clinical applications, tDCS has an advantage over rTMS in terms of cost-effectiveness.

## Figures and Tables

**Table 1:** Demographic data of enrolled patients.

	rTMS	tDCS	*p*-Value
sex (male:female)	21:15	17:16	0.572
age (years old)	56.1 ± 12.3	59.3 ± 11.2	0.264
side (right:left)	18:18	16:16	1.000
worse ear PTA	38.9 ± 24.3	32.3 ± 23.5	0.233
better ear PTA	19.7 ± 12.8	16.4 ± 10.0	0.356
pre-treat VAS loudness	7.4 ± 2.0	7.1 ± 1.8	0.482
pre-treat VAS awareness	7.6 ± 2.0	7.6 ± 1.9	0.976
pre-treat VAS annoyance	6.7 ± 2.1	5.9 ± 2.5	0.248
pre-treat THI	53.2 ± 22.9	50.0 ± 19.8	0.639

**Table 2:** Comparison of treatment results between rTMS and tDCS.

	Immediately After			1 Month After		
	rTMS	tDCS	*p*-Value	rTMS	tDCS	*p*-Value
VAS lo	6.3 ± 2.4	6.8 ± 2.0	0.517	6.1 ± 2.0	6.1 ± 1.8	0.990
VAS aw	6.7 ± 2.1	7.2 ± 2.1	0.276	6.4 ± 2.2	6.1 ± 1.8	0.493
VAS an	5.8 ± 2.4	5.6 ± 2.5	0.956	5.9 ± 2.7	5.0 ± 2.2	0.131
THI	46.1 ± 23.8	47.5 ± 23.3	0.714	42.2 ± 19.6	40.6 ± 20.4	0.745
Improvement ratio ^1^	11/23/2	4/29/0	0.054	17/17/2	12/18/3	0.618

^1^ “Improvement rate” values indicate improvement/no change/worsening.

## Data Availability

Data sharing not applicable.
